# Beyond the urban shift: towards a relational degrowth spatial politics

**DOI:** 10.1007/s11625-024-01590-7

**Published:** 2024-12-12

**Authors:** A. Varvarousis, G. Kallis, C. Catanneo, F. Sekulova, M. Tsagkari, A. Slamersak, M. Conde, G. Dalisa, K. Hanacek, B. Roy

**Affiliations:** 1 Institute of Environmental Science and Technology (ICTA), Autonomous University of Barcelona (UAB), Barcelona, Spain; 2 Department of Environmental Studies, Faculty of Social Studies, Masaryk University, Jostova 10, Brno, Czech Republic; 3 Department of Geography, Autonomous University of Barcelona, 08193 Bellaterra, Spain; 4 Faculty of Economics and Business, Universitat de Barcelona, Barcelona, Spain; 5 IN3, Universitat Oberta de Catalunya, Barcelona, Spain

**Keywords:** Degrowth, Urban studies, Urban–rural relations, Urbanization, Planetary urbanization, Depopulation, Utopianism, Post-growth planning

## Abstract

Degrowth is coming of age, and its analysis of what can be called *degrowth spatial politics* is advancing rapidly. In this article, we attempt to unravel the history of spatial thought in the degrowth literature to reveal its tendencies, tipping points, and blank spots. We argue that the more recent degrowth spatial literature overly focuses on the big urban scale by reversing what has purportedly been one of the weak spots of degrowth scholarship: its focus and preference for small-scale, relocalization, and decentralized communities. While we see the merit of this shift, first, we try to contextualize it in the broader sustainability discourse, and second, we contend that it has not been without problems and omissions. We see three predicaments stemming from it: first, a deficit of the degrowth spatial literature in recognizing and engaging with unsustainable rural transformations such as rural depopulation and shrinkage, as well as rural dispossession and depeasantisation. Second, a difficulty to account for urban and rural interconnectedness and engage with new epistemological frames that emerge in urban studies, such as planetary urbanization. Third, the urban shift may affect the capacity of degrowth to remain pluriversal, anti-colonial, and grassroots-fueled, or what Chertkovskaya et al. (Towards a political economy of degrowth. Rowman & Littlefield, 2019. https://books.google.com/books?hl=en&lr=&id=PuXaDwAAQBAJ&oi=fnd&pg=PP1&dq=info:c45bQLgYCfYJ:scholar.google.com&ots=5LsDF9Y_Er&sig=buZn4ogpUN4octi3_WFJf1mO9Lg) called *nomadic utopianism*. Finally, in the concluding part, we set the stepstones for a relational degrowth spatial politics, focusing on a solidary connection of space and place across the urban and rural and in multiple scales. This approach avoids the pitfalls of both degrowth proposals for relocalization and those who put excessive trust in urban transformations alone.

## Introduction

Degrowth is a fast-growing area of interest and research. The literature on the spatial organization of degrowth, in particular, is flourishing, shedding light on this crucial but neglected aspect of degrowth transition. Some form of spatial awareness has undoubtedly accompanied degrowth since its early stages; however, we are witnessing now a two-dimensional evolution that could be framed as *degrowth spatial politics*. Degrowth scholars have begun to engage with the fields of urban and regional planning (Kallis et al. 2020), urban and rural studies (Gerber 2020), geography (Demaria et al. 2019), and development studies (Kallis et al. 2022) on the one hand and, on the other and using a degrowth lens, urban planners and geographers are rethinking their sustainability-oriented research and policy agendas (see for example Kaika et al. 2023; Krähmer 2022; March 2018; Savini 2021; Savini et al. 2022; Xue 2014; Xue and Kębłowski 2022).

By historicizing degrowth spatial thinking, we reveal a tendency in recent contributions to privilege the urban sphere over the rural. In this analysis, we attempt to explain this focus on the big urban scale by reversing what has purportedly been one of the weak spots of degrowth scholarship: its focus and preference for small-scale, relocalization, and decentralized communities (Krähmer 2022; Mocca 2020; Xue 2014). We agree with the diagnosis that degrowth spatial politics should not be limited to relocalization, ecovillages and bioregions, and we endorse the expansion of the degrowth literature towards the urban realm. Nonetheless, we contend that this shift has not been without problems and omissions. First, we claim that the shift towards the urban is not coincidental but inscribed into the broader transition in sustainability science and policy in which cities are no longer framed as a problem for planetary sustainability but as the solution to the problem. To this end, we follow Angelo and Wachsmuth (2020: p. 2202) who ask, " Why does everyone think cities can save the planet?" and develop their critique against this trend. They argue that urban sustainability discourses cannot revolve around livability, density, green technology, and resilience only; they promote a different research agenda with historical, political, multispatial, and representational elements. Our analysis builds on this approach, arguing that a focus on cities as privileged sites for implementing degrowth spatial politics allows only a very narrow framing of socio-environmental problems and solutions. This ignores crucial questions for degrowth that include: rural shrinkage and abandonment, urban–rural relationality, and the subjugation of the countryside to cities; the impacts of urban greening to rural areas; the intensification of cost-shifting from the Global North to the Global South, specifically through a city-centric sustainability discourse and policy; and, finally, privileging a top-down research agenda and policy at the expense of a pluriversal degrowth "nomadic utopianism" (Chertkovskaya et al. 2019).

In our view, just and effective degrowth spatial politics should involve a wholly different approach to urban–rural relations based on *delinking and restructuring the harmful spatial relations of "planetary urbanization*" (Brenner and Schmid 2015) *and establishing different, reciprocal connections in a degrowth spirit between urban cores and rural peripheries, the Global North and the Global South.* We envisage this modality of thinking about degrowth spaces as acknowledging the relationality of space and avoiding a purified conception of degrowth as a rural, urban, or other monolithic project that privileges certain spatial units of analysis over others (Spanier and Feola 2022). This conception brings flows, dependencies, and power geometries into the picture and enables a way of thinking and planning about degrowth that strengthens local sovereignty, autonomy, and sociocultural diversity without resorting to simplistic, formalistic proposals.

This paper is structured as follows. The first section offers a comprehensive review of degrowth spatial thinking that explains how cities gradually replaced relocalization, eco-communities, and other formalistic anti-urban proposals to dominate discussions around the spatial forms of a future degrowth society. This part follows a historicizing approach that, in contrast to earlier comprehensive reviews of the field (Kaika et al. 2023; Krähmer 2022), does not aim to create typologies or identify gaps in the literature. Instead, it seeks to reveal tendencies, tipping points, and pulling factors that bring about these changes. The second section analyses the implications of this tendency towards the urban and details its shortcomings and pitfalls. It also highlights its negative impact and brings the rural perspective more into focus. The paper concludes with a discussion of the main areas for future research on the topic.

## Section 1: from horizontal to vertical: how degrowth spatial politics moved from the forest to downtown

### Early utopias of the spatial form

Degrowth originated in France in the 1970s. André Gorz first introduced the term *décroissance* in a public debate where he questioned the compatibility of the survival of capitalism with environmental sustainability (Gorz 1972). This early call for degrowth was followed by political and philosophical discussions on a series of degrowth-related issues in the francophone world. These included ecology, self-limitation, and autonomy (Castoriadis and Cohn-Bendit n.d.), anti-utilitarianism (Latouche, Caillé and other members of the MAUSS-Mouvement Anti-Utilitariste dans les Sciences Sociales), and critiques of techno-optimism and science-centered solutions. These discussions became an emergent social movement after 2001 with Lyon (France) as its epicenter (Demaria et al. 2013). The popularization of the term "degrowth", initially construed as a "missile word", a "semantic bomb", or an "explosive term", aimed at disturbing the post-political consensus around the imperative of economic growth and its central role in human and societal development. Degrowth advocates subsequently combined this use of language with a similarly provocative and disruptive repertoire of actions. One notable example is Francois Schneider's walking tour of France accompanied by a donkey and spreading the word of degrowth (Demaria et al. 2013). Another was Enric Duran's bike tour for degrowth in Catalonia, through which he acquired bank loans totaling half a million euros that were never intended to be repaid. His intention was to visibilize how the banks never paid for the 2008 financial crisis themselves, despite being its main cause (D’Alisa et al. 2014). Further actions were the resistance to eviction and the radical experimentation with frugal lifestyles of the green anarchist squatters of Can Masdeu in Barcelona (Cattaneo and Gavaldà 2010), community-based initiatives oriented towards forms of provisioning that prefigure degrowth (Sekulova et al. 2023), and the degrowth remote island communes of the Greek archipelago (Varvarousis 2012; Kallis et al. 2022).

This provocative spirit continued in the degrowth literature in its first attempts to consider degrowth spaces. Indeed, early degrowth scholarship imagined the spatial form of the degrowth society to be a network of interconnected ecovillages, communes, urban villages, or self-sufficient bioregions (Bonaiuti 2012; Homs 2007; Latouche 2009; Alier 2009; Trainer 2000, 2012; Varvarousis 2012). The Australian school of degrowth, the so-called "Simpler Way", is central to this approach, consisting of scholars who have long argued in favor of radical relocalization and a degrowth spatial organization around self-sufficiency and smallness (Martinez-Alexander and Gleeson 2019; Alexander and Rutherford 2020; Schneider 2018; Nelson 2018; Trainer 2000, 2006, 2012) and explored broader policy and urban planning strategies (Dodson et al. 2017). Degrowth thinkers in Europe, such as Latouche (2009, 2004), focused more on the spatial image of older Mediterranean cities and towns. This focus is exemplified in *La Utopie Méditerranéenne* that argues for a "meridian alliance" between the countries of the European South (Latouche 2008) based on the values of moderation, slow living, and conviviality (Cassano 2012). Relocalization, however, remains at the heart of the degrowth transition, defined as a massive departure from cities to form small, self-sufficient settlements within which localism may have become a universal solution (Krähmer 2022; Mocca 2020).

These initial efforts to imagine degrowth spatialities invoked mainly spatial images opposed to the idea of an urbanized world defined by its metropolises and their polluting complexity; its "global cities" in Saskia Sassen's (1991) terminology. These images are symptomatic of a discourse and political movement that aims to provoke and destabilize. For Harvey (2000), the "utopia of the spatial form"^1^ is the primordial scheme for seeking alternatives: it is easy to juxtapose with the existing order, easy to comprehend, and also points towards a future of harmony and stability, easily contrasted with the chaotic present. Theoretically, these utopias can be tried here and now without the prerequisite of a slow and complex systemic change, because they seek independence from that order. A further reason for the early degrowth scholarship focus on such utopias as ecovillages, urban villages, or bioregions, is the absence of scholars from geography, urban studies, and urban and regional planning. The majority of degrowth publications until 2015, including the first five degrowth-related special issues, attempt to provide a fundamental ontology of the concept and to explain why it is needed (D’Alisa et al. 2014; Demaria et al. 2013). These early publications sought to debunk the "myth of decoupling" and the narrative of sustainable development and green growth (Kallis 2011; Schneider et al. 2010), to link degrowth with political ecology and environmental justice (Martinez-Alier et al. 2016; Martínez-Alier 2012), and to explore the politics of a degrowth transition (Cattaneo et al. 2012; D'Alisa et al. 2014). Of course, it is evident that new spatialities must and will emerge, since degrowth argues for a profound socioecological shift to a postcapitalist society, as the next section outlines, but given the absence of urban scholars and geographers in the early phases, these questions remained scattered and marginalized.

### The urban shift

The shift in degrowth thinking with regard to urbanization and a broader scholarly interest in degrowth is evident in the chronology of publications. The first publication on degrowth by an urban scholar occurred in 2014. Xue (2014), an urban planner, essentially shifted the locus of interest from small-scale, primarily rural, self-sufficient settlements, such as eco- and urban villages, to centralized urban planning strategies. Xue critiques what she perceives as a one-dimensional approach to degrowth spatialities based on economic, political, and ecological relocalization. She argues that the majority of the earth's population will not desire such massive relocalizations. In her view, such a shift would eliminate the amenities now typically provided by urban cores, would demand an increase in energy and raw materials, and would produce extensive waste (due to abandoning urban infrastructures). Furthermore, whether such a shift would deliver the desired outcomes, such as solid community bonds, enhanced autonomy and democratic capacity, or ecological sustainability, is far from certain.

These critical insights marked a broad turn in degrowth interest towards cities and urbanization processes, although the engagement of urban scholars and geographers with degrowth remained limited until the end of the 2010s. Kallis and March (2015) published an article on multiscalar degrowth a year after Xue's article. Their article, inspired by Le Guin and also grounded in the example of the *Cooperativa Integral Catalana* (CIC), argued in favour of "a new politics of scale" to overcome the bounding of degrowth to the small scale and to subsequently rescale relations of care, production, and social reproduction in multiple levels. However, this work did not increase interest in degrowth in urban studies scholarship nor in connecting degrowth to urban sustainability agendas. A few articles were only published, marginal to degrowth and urban studies, relating to urban shrinkage (Schindler 2016) or repeating the logic of relocalization and utopian thinking (as above, Latouche 2016).

The coupling of the dialogue between degrowth and critical urban studies occurred only in 2019. Several events signify this connection. First, the Oslo Architecture Triennale entitled "*Enough. The Architecture of Degrowth*" marked the increased interest of architects and urbanists in degrowth, producing at the same time a series of perspective pieces on the implications of a degrowth transition for urban planning and architecture (see, for example, Varvarousis and Koutrolikou 2018). Second, Demaria, Kallis and Baker (Demaria et al. 2019) published a special issue in the same year, entitled "*Geographies of degrowth: Nowtopias, resurgences and the decolonization of imaginaries and places*", which marked the shift of degrowth thinking from utopias of the spatial form to *spatiotemporal processes* for degrowth transformations. This special issue included multiscalar degrowth strategies based on case studies ranging from the Mexican Chiapas, South India, Athens, Turkey, and British West Essex. The same year, Nelson and Schneider’s “Housing for degrowth” volume hosted a pertinent debate between scholars on the intersections of housing, urbanization, and planning. A common denominator in this debate was the critique of (re)localization and the so-called “local trap” (Purcell 2006). Xue (2018) explained and deepened her previous position on the impossibility and undesirability of localization, decentralization of politics and absolute egalitarianism. Vansintjan (2018) criticized urbanization as “the death of politics”, discussed Bookchin’s municipalism, and reframed the debate upon what he calls degrowth municipalism—the “politicisation and downscaling of municipalities’ social metabolism necessary to achieve a more just society, will require the creation of an organic citizenship or the citification of urban space” (ibid: 294).

Subsequent publications attempted to link degrowth and urban planning. In 2022, Xue and Kębłowski edited a special issue (SI) entitled "*Spatializing degrowth, degrowing urban planning*" to help reinvent urban planning and to explore how degrowth could be concretely spatialized in cities. The entire SI was a call to "take cities more seriously". It expanded Xue's previous work on compactness, reduction of space per capita, and redistribution of urban resources (Xue 2022; Xue and Kębłowski 2022). The SI also explored the application of these principles in different realms, such as in mobility through less space for cars, high-density, low-carbon infrastructures, and shared mobility options (Cattaneo et al. 2022; Chertkovskaya and Paulsson 2022; Ruiz-Alejos and Prats 2022), and in housing through per capita capping for housing consumption, reduction and optimization of housing space, refurbishing of old housing stocks, and redistributing housing stock through, for example, collaborative and cooperative housing schemes (Cucca and Friesenecker 2022; Martínez Alonso 2022; Mete 2022).

Savini et al. (2022) edited a book on "Post-growth Planning: Cities Beyond the Market Economy" in the same year in which they examined the various properties of a degrowth city partitioned into categories such as dwelling, nurturing, governing, and moving. They synthesized their findings in a "Manifesto for Post-growth Planning" articulated along six axes: residential buildings to enhance community and reduce environmental impacts; transport systems to prioritize socioecological sustainability; institutions that, instead of protecting private property, seek to pursue autonomy and democracy; planning institutions to increase social value rather than land prices; providing resources to foster human health while reducing waste; and redirecting planning ethics from a culture of competition towards one of trust and compassion.

Lange et al. edited a book, also in 2022, on the *Post-growth Geographies* that pushed the discussion of spatial politics further through additional case studies (mainly urban) and, crucially, by enriching the degrowth conceptual panoply through explaining and operationalizing concepts from the field of geography, such as scale, network, territory and place, terrain, landscape and border. Furthermore, the book advances degrowth spatial literature in different directions, including in architecture and design, degrowth spatial innovation, post-development, and more, by following a nonlinear structure to compile perspective papers, interviews, and practical examples.

Finally, Kaika et al. edited a further SI in 2023 entitled "*Urbanizing degrowth: towards a radical spatial degrowth agenda for future cities*". The focus here is not on cities but on urbanization processes. The editors paid particular attention to the Global South/Global North relations while trying to imagine an open process, broken down into concrete steps, about moving from growth-based urbanization towards diverse post-growth futures. These steps include: historicizing spatial degrowth experiments to create know-how for the future; developing a critical dialogue with planning institutions at all levels to translate degrowth agendas into concrete realities; forging urban and regional alliances for upscaling existing degrowth ventures without co-option; engaging urban professionals in everyday insurgent degrowth practices; recognizing and prefiguring uneven and unequal spatial degrowth outcomes for different regions in the Global North and South. The special issue is possibly the first attempt to organize the spatiotemporal process of degrowth transformation, not in projected spatial units and institutions, but in open-ended strides.

Lehtinen (2018) problematized the compact city model and other contemporary planning imperatives from a degrowth perspective before the publication of these major works by focusing on Joensuu, a city in Eastern Finland. He finds that, in contrast to dominant narratives, compactness is often associated with changes in consumption patterns that increase levels of carbon emissions that, in turn, put more pressure on the surrounding countryside and peri-urban nodes. Ferreira and Schönfeld interlaced urban planning with degrowth in 2020, a prelude to their edited volume described earlier. Savini (2021) explored the mechanisms that bind urban planning to economic growth, identifying enforced land scarcity, zoning, and regional territorialization as the primary drivers. Subsequently, he identifies degrowth terms for these mechanisms, such as finity, habitability, and polycentric autonomism. Finally, Krähmer provided a systematic recap of the existing "degrowth and the city" literature in 2022, pointing out that much was still based on localism. While he finds degrowth critiques of mainstream green growth agendas compelling, he points to a significant gap in how it deals with urban material flows and bigger scales. He also problematizes degrowth engagement with theories of space production and positionality.

This apparent shift towards cities applies not only to degrowth publications with an explicit geographical, planning, or spatial focus but also to recent degrowth books. In her reading of the books "*The Future Is Degrowth*" (Schmelzer et al. 2022), "*Degrowth & Strategy*" (Barlow et al. 2022), and "*The Case for Degrowth*" (Kallis et al. 2020), Gasparro (2023) noted that the terms *urban*, *city*, and *cities* combined appeared almost ten times more frequently than *rural* or *countryside*.

A handful of papers only are notable recent exceptions to the above trend. Gerber (2020) brings the "agrarian question" into the picture, explaining how Critical Agrarian Studies can and should develop vis-à-vis degrowth. Ertör and Hadjimichael (2020) explore the merits of what they call "blue degrowth" to repoliticize the seas and oceans to explore the multiple dimensions of the degrowth politics of the sea. Kallis et al. (2022) also focus on what they call "adeveloped communities of the Greek archipelago". They regard these rural societies as "real-existing degrowth", not because degrowth has arrived there but because they identify imperfect and incomplete processes in these societies of resisting growth or adapting to its end through tentative alternatives. Finally, Spanier and Feola (2022) specify this urban turn explicitly in the degrowth literature, arguing to abandon the rural–urban dichotomy and stressing urban and rural relationality.

### Drivers of the urban shift

Critical questions arise from this conceptual evolution. How did spatial degrowth scholarship make this u-turn from ecovillages, urban villages and self-sufficient settlements to the (compact) city and urban agendas and why was it done? What are the implications of such a shift? We argue that the shift is not exclusive to the degrowth literature but results, at least partially, from parallel trends in broader sustainability discourses and agendas. As Angelo and Wachsmuth (2020) argue, there has been a steady transformation in sustainability science during the last decades whereby cities are no longer viewed as a sustainability problem but as a solution to sustainability problems.

Three historical developments explain this shift. The first concerns a reaction to urban sprawl and advocacy for a compact view of city development (Jenks et al. 1998). In the US, where the sprawling model of urban development was dominant and took its most extreme form, the continuous expansion of suburbs was viewed as the ultimate source of unsustainability (Angelo and Wachsmuth 2020). However, once elites and the middle class began to reinhabit urban centres in the 1990s and early 2000s, the "compact city" concept was consolidated as an alternative to urban sprawl. Thus, urban development had a double connotation: the suburbs continued to be associated with unsustainability on the one hand and, on the other, dense urban centres began to figure as a sustainable solution. In its more extreme form, this sustainability discourse, which is based on vertical cityism and density, features some of the most provocative (and perhaps monstrous) ideas of contemporary architecture. Young's "Planet City" (Young 2021) is possibly the best example of this compact urban utopia: a project of "an imaginary city for 10 billion people who have surrendered the rest of the world to a globally scaled wilderness and the return of stolen lands".

The second development driving the sustainability discourse to embrace cityism relates to the explosion of urban slums in the Global South (Angelo and Wachsmuth 2020). Historically, urban slums were listed as environmental problems, similar to cities in general, due mainly to their polluting effects on their inhabitants and surroundings (UN-Habitat 2003). However, the urban slums of the Global South began to be more positively regarded due to their acute density, informal economic networks, informal recycling practices, sense of community, walkability, and economies of scale (Angelo and Wachsmuth 2020). This compact city narrative became stronger when combined with the emerging discourse of urban resilience.

The third development driving the sustainability shift towards city-based solutions is climate change. When a changing climate was initially framed as an emerging sustainability problem in the 1980s and early 1990s, cities were viewed as the main drivers of greenhouse gas emissions, similar to the other two drivers. This was due, first, to their increasing population and, second, to the unsustainable consumption and lifestyle choices of wealthier residents (Angelo and Wachsmuth 2020). Gradually, as urbanization began to be seen as an irreversible trend, more effort was invested into making cities "climate-proof". This pendulum change was facilitated by the emergence of the "resilience" discourse, fabricated by mainly urban dwellers to protect urban areas. It played a pivotal role in shifting urban planning mechanisms towards measures to mitigate the effects of climate change. Second, city leaders globally have increasingly adopted a "responsibility" narrative about the role of cities in fighting climate change, leading to the creation of numerous city networks and coalitions, such as the C40, the Fearless Cities, the Global Covenant of Mayors for Climate and Energy, and others. However, these coalitions were also promoted as "antidotes" to inaction on climate change at the national level and the election of climate change deniers in major emitting countries.

Even though these reasons drive sustainability discourse and policy towards the urban, several reasons endogenous to degrowth scholarship specifically further amplified this trend. Degrowth is gaining momentum both at the scientific and policy levels. Once a marginal and provocative literature and social movement, degrowth is now being discussed at the highest levels in the European Parliament, the UK House of Commons, and many other policy-oriented venues. Such heightened publicity, combined with the increasing emergency of the exploding climate crisis, unavoidably pushes degrowth beyond merely assessing the problem or sketching tentative sociocultural solutions. Degrowth scholarship is now expected to provide concrete answers and robust policy solutions. The EU is funding numerous research proposals for degrowth with prestigious ERC grants.^2^ This impacts on the research agenda and the nature of degrowth research. Thus, a more feasible pathway than the extensive and somewhat utopian relocalization thesis of early degrowth scholarship is acknowledged in accepting urbanization, focusing on regulating it, making it compatible with a degrowth transition, and lobbying at the top policy level to push such change through.

This strategy comes at a great cost, notwithstanding its appeal. There is strong evidence that current urbanization patterns are incompatible with a degrowth transition to which we turn in the next section.

## Section 2: the sacrifices of the urban turn

### Inability to recognize unsustainable rural transformations

The degrowth literature contains much that is both in favor of or rejects relocalization in rural areas, mainly from a normative perspective. There is, however, a significant gap in connecting growth with unsustainable transformations in the rural world. Core drivers of global unsustainability and sociospatial inequality have been identified to date as depeasantization, rural abandonment, settlement shrinkage and depopulation, and the transformation of non-urban land into sacrifice zones. None of these phenomena have been adequately examined from the perspective of degrowth spatial politics. *We argue that this is a missed opportunity, since degrowth is a framework that can offer substantial solutions to these growth-driven destructive processes.* Notable exceptions are recent master theses that investigate ¨Revaluing the rural¨ (Gasparro 2023) and the contribution of degrowth architecture to countering rural depopulation (Sokolovskyi 2022). This identified gap further strengthens the argument of the present paper. Early degrowth literature employed a primordial form of spatial thinking. This conceived the world as a tabula rasa, to be redesigned on the principle of radical smallness (see also Krähmer 2022) with sketches of hypothetical "simpler way" communes. The latest stream of research in the field has shifted the focus to cities as the primary locus of degrowth spatial politics, in line with other sustainability discourses. The time is now ripe for degrowth to engage with existing rural areas and the challenges they face.

These rural transformations have been extensively studied outside degrowth. Global depeasantization, understood by agrarian scholars and rural sociologists to be the gradual and systematic assault on and dismantlement of rural, communitarian, and agrarian forms of sociality (Arboleda 2020), constitutes the "great global enclosure of our times" (Araghi 2000). Its spatial expression takes the form of "global deruralisation^3^" and "global hyperurbanisation" (ibid). Hobsbawm (1994) goes further, arguing that depeasantization was the most fundamental change of the twentieth century, even above events such as the two world wars, decolonization, the IT revolution, and the rise and fall of state socialism.^4^ The phenomenon takes diverse forms with different push and pull factors driving it in different geographies and at different temporalities. It remains globally significant. Araghi (2000) estimates that 65 per cent of the growth in urban populations in the 1980s and 1990s was attributable to rural–urban migration. Gosh and Meer (2020) assert that global push factors are related to complex operations, such as surplus dumping via food aid programs, growing food import dependence, and exposure to international price forms. Global pull factors are related to developmentalist 'modernization' that draws rural populations to metropolitan regions by promoting domestic industrialization and urban development (ibid).

A constant trend of rural depopulation and abandonment has been evident in Europe since the 1960s that is uneven across both countries and times (Raugze et al. 2020). Europe has the lowest rural population in the world overall, with only 20.6% living in rural areas (Eurostat 2023). Additionally, there is the prospect of losing a further 7.9 million people up until 2050. This contrasts with urban areas that are expected to grow by up to 24 million people over the same period (Raugze et al. 2020). In some eastern and southern European countries, more than 80 per cent of rural areas are shrinking (ibid). The reasons vary. Collantes and Pinilla (2011) argue that in Spain, for instance, which is among the most affected of EU countries in terms of depopulation, the "peaceful surrender" happened in the twentieth century through processes of industrialization and mechanization in agriculture, pro-urban public policies and uneven allocation of funding, transformation of lifestyles, and changes in non-farming rural sectors.

Depeasantization advanced in less peaceful ways in other parts of the world: through entangled cycles of enforced displacement and dispossession (Arboleda 2020) and the so-called "planetary landgrabs" (Araghi 1995). In Latin America, the explosion of manufacturing enclaves, special economic zones, agroindustrial hinterlands, and open-cast mines in countries such as Chile and Colombia would be impossible without the dispossession of its peasantries (Arboleda 2020). In Colombia, for instance, agroindustrial elites and paramilitary groups forcibly displaced about six million people from their rural homes in recent decades (Hylton 2006). In Asia, China's economic miracle would not be possible without transforming its peasantry of hundreds of millions into urbanized industrial workers (Arboleda 2020). In India, Roy (quoted in Arboleda 2000) describes forced rural–urban migrations involving elites, the military, and even "death squads" responsible for the deaths of approximately 250,000 people, either by suicide as a means of fleeing the horrors of punishing debt or through harsh proletarianization and even hunger.^5^ More recent research (Zografos and Robbins 2020) suggests that depeasantization can occur even through the implementation of "green sacrifice zones" as part of green growth agendas that can reproduce colonial relations in the name of "just transition".

The impact of rural dispossession and depeasantization are severe in both social and biophysical spheres, although their effects are diverse, depending on the region and its former land use as well as on various biological and morphological factors. First, in the biophysical sphere, rural abandonment is often associated with modifying the hydrologic and biogeochemical cycles and reducing biodiversity (Seto et al. 2012) while increasing, at the same time, soil erosion, the possibility of forest fire, and invasions by pests and weeds. This, in turn, affects the diversity of natural habitats (MacDonald et al. 2000; Lasanta et al. 2006; Alomar and Bardi 2007; Joy and Medrano 2007). In flat and fertile lowlands, particularly those with water reserves, rural depopulation is argued as paving the way for unsustainable land use intensification and ecological degradation through industrial farming and monocultivation (Tscharntke et al. 2012). This has led to the loss of local varieties of fruits and vegetables. The underlying assumptions in many green proposals that celebrate urbanization, urban density, and the separation of nature from the human milieu for conservation reasons are contested within the conservation community for both their ineffectiveness and their severe adverse consequences on societies and their environments (Büscher and Fletcher 2020).

Second, in the social field, depeasantization, rural depopulation and land abandonment have equally severe consequences for the remaining rural populations and those who live in the cities. The gradual erosion of rural practices and livelihoods, the loss of local, situated knowledge, and the displacement of traditional resource management systems increase unsustainable growth dependencies at all scales. The enforced or incentivized depeasantization impacts on the way 'the rural' is framed and valued, especially for remaining young rural population groups, with pejorative connotations of backwardness and inferiority, fueling further depopulation tendencies and a vicious cycle of land degradation (Llorent-Bedmar et al. 2021). Within this cycle, quality of life decreases for the remaining rural population, since access to essential services (e.g., health, education, cultural recreation, etc.) becomes scarce and costly, the population ages, and rural areas lose further economic dynamism. Finally, more women abandon rural areas than do men, because the employment options are fewer, and rural environments are traditionally more oppressive, leading to a phenomenon termed *rural masculinization* (Camarero and Oliva 2019).

Usual policy responses to rural shrinkage can be divided into three types: the first is to deny that rural depopulation is a problem and instead to accelerate current urbanization trends (Raugze et al. 2020); the second is to reverse the trend and stimulate rural population growth; the third is to accept the trend and try to adapt to demographic changes (Raugze et al, 2020)—to "shrink smartly" as the suggests^6^. However, there is strong evidence that even in cases where governments take serious measures to mitigate or reverse rural depopulation and abandonment trends, they are failing. Almonte and García (2016) and Bayona-i-Carrasco and Gil-Alonso (2012), for instance, showed that, despite policy attempts to counteract depopulation in Spain over the last two decades, most rural areas continued to experience population declines. Other scholars (e.g., Bätzing et al. 1996 for the Alps) have shown how contemporary urbanization patterns combined with policies to counter rural population decline have fueled the production of uneven geographies and contributed to the further disintegration of previously connected areas.

We argue that existing strategies have little chance of success without the dogma of economic growth being challenged. Depeasantization, depopulation, and abandonment are driven by global capitalist accumulation and growth extraction trends founded on subjugating the rural to the urban by creating more operational landscapes and sacrifice zones. Green and smart growth agendas only intensify the problem by adding more nodes to this dense global network of value extraction. Degrowth can offer solutions to these problems by decoupling human prosperity and economic growth and advocating for a decolonial, equitable, and solidary interconnection of place and space. Degrowth can offer a revalorized view of the rural in the peri-urban sphere of the struggle for peasantry on the verge of the urban (Sekulova et al. 2023) or cases of real-existing degrowth and adeveloped communities (Kallis et al. 2022), by connecting with local resistance against rural depopulation (Paniagua 2017) and simultaneously avoiding idealization.

### Failing to account for urban and rural interconnectedness

Much of the debate around the spatial organization of degrowth has been played out over the dipole city vs rural. There has been little reflection, however, about what constitutes the urban, the rural, and the spatial configurations in-between. Our argument here is that this deficit in appreciating interconnectedness substantially impacts on the capacity of degrowth scholarship to articulate a balanced understanding and strategic program for its spatial future beyond *urbanophilia* and *urbanophobia* (Félonneau 2004). It is a truism that the urban–rural divide is nothing new. In the nineteenth century, as Angelo (2017) notes, a spatial understanding based on binary interpretations of the world developed whose fundamental assumption was that a city can be defined in juxtaposition to a non-urban. This became evident with the emergence of the first big industrial metropolises, for example, London, New York, and Paris—and their so-called new "folk". This folk "city lens" gave birth to a new epistemology of the urban that has been uncritically adopted, and continues to define, the broad fields of urban planning and urban studies (Angelo 2017; Brenner and Schmid 2015). The fundamental characteristics of this epistemology are: (a) understanding the world through binarism (urban/rural, agrarian/industrial, modern/traditional, etc.); (b) a fixation on self-contained spatial units (the city, the town, the village, etc.) that also function as units of analysis; (c) the adoption of generic universal metrics for defining and distinguishing the urban from the non-urban (e.g., population size, density thresholds, administrative borders, etc.); and (d) the conceptualization of the urban through a series of axiomatic, universalistic properties, such as cosmopolitanism, political progressiveness, service-based economies, and more.

Several problems stem from this city-centric epistemology. First, it does not grasp how urbanization processes reshape both the city and the "hinterlands". These become the operational landscapes of spatial development under extended urbanization, or planetary urbanization in Brenner and Schmid's (2015) terminology. Second, keeping the city and the rural realms separate, or contrasting them, promotes a fragmented understanding of the politics of change. In this vein, several studies have attempted to calculate the ecological footprint of the city vis-à-vis rural dwellers (Heinonen and Junnila 2011; Pang et al. 2019). Most, while using elaborate techniques to perform these calculations, nonetheless fail to take into account cost-shifting between the cities and the country (Dorninger et al. 2021; Conde et al., 2022), unequal ecological exchange (Dorninger et al. 2021), and land transformations that occur far from city centres. This failure brings with it misleading results and interpretations of the sustainability of the urban and the rural. Furthermore, the rural/urban dichotomy is the backbone of all design-oriented greening solutions that, even from the era of Howard's Garden Cities, try to resolve the city's "grey urban nature" impasse (Wachsmuth and Angelo 2018). The mechanisms used, for example, the development of green areas and infrastructure or "nature-based-solutions" (Sekulova and Anguelovski 2017), thus neglect the fact that urban unsustainability is not fueled by what happens in urban areas alone. Third, the universalistic language of the city-lens epistemology is rooted in a colonial understanding of the urban and urbanization, since it extrapolates a specific and historical urban form, i.e., the Western industrial city and its properties, to operate as a model for what a city is and is not globally and temporally.

Urban studies have long insisted on a binary urban epistemology based on separate spatial units (Angelo 2017), even though the metabolic relation between cities and hinterlands has been known since Marx's theory of the spatial (metabolic) rift (Saito 2023). However, in the last decade, and amid the peak of the so-called "urban age", a series of new epistemologies seeks to deconstruct and reframe the "urban question" (Castells 1972). Brenner and Schmid's planetary urbanization is central to this effort. They argue that even those spaces lying well beyond the traditional city core have become integral parts of the worldwide urban fabric (Brenner 2018; Brenner and Schmid 2014, 2015). They articulate their eloquent analysis in a series of critical interpretations of the existing "old and new urban ideologies", including "urban triumphalism", "technoscientific urbanism", "megacities", and "city sustainability" discourses. They formulate a proposal for a "new epistemology of the urban", which, similar to other post-colonial formulations in different fields, emphasizes the relationality of space production between the city core and the hinterland. This invites us to think in terms of urban transformations—and so to avoid "methodological cityism" (Angelo and Wachsmuth 2015).

Brenner and Katsikis (2020) extended the theory of planetary urbanization more recently by suggesting four critical mutations in city/hinterland relations. First, they observe the phenomenon of "distanciation and infrastructuralization", meaning that cities are no longer fed by their adjacent hinterlands but through global, transnational networks. At the same time, this type of productivity is based on colossal infrastructural projects creating monofunctional landscapes that transform rural areas into "sacrifice zones". Second, as global supply chains expand to form an "intercontinental logistics space", rural areas are losing their direct relation to specific cities, becoming "hinterlands of hinterlands" and complicating the nature of global production and commerce as well as their transformational processes. Third, as the margin for extending resource and commodity frontiers narrows from the overexploitation of a finite world, new technoextractive logics and apparatuses are employed in the hinterlands to transform them into global territorial ecological machinery. Fourth, as the environmental surpluses of the earth are diminishing, new and deeper metabolic rifts are being created. These cycles of "creative destruction" give rise to extended and escalating conflicts over the future of these landscapes.

New challenges in the degrowth project are arising from spatial relationality and recent changes in the city/rural relations. Degrowth spatial transformation can thus no longer be conceived as an urban, rural, or other monolithic project. One can find only limited responses to the challenges by looking at the tentative responses of urbanists to these new conceptualizations and understandings of the spatial world. Angelo (2017) proposes hybridity to overcome binary urban studies scholarship, following insights from urban political ecology, post-colonial urban studies, and American sociology. She examines the limitations of this effort, offering instead to turn dualisms into research objects through the study of urban imaginaries, histories, and discursive transformations (ibid). More recently, Angelo and Wachsmuth (2020) extended this research agenda to emphasize the need for a historical, multispatial, political, and representational analysis of urban sustainability thinking and policy. Brenner and Katsikis (2020), the very theoreticians of "planetary urbanization", and despite their eloquent criticism, offer no concrete exit and end their analysis by asking a series of essential questions on the nature of hinterlands in the capitalocene.

Very little engagement with the topic of deurbanization has occurred from a degrowth perspective. A notable exception includes Spanier and Feola's (2022) work on "nurturing the post-growth city". They advocate "bring(ing) the rural back" into city planning by unmaking the urban/rural cultural rift, hybridizing both realms, and "liberating" the rural from urban subjugation. The authors suggest four elements in future research to do so. First, they call for an acknowledgement of the existing performances and concrete geographies that challenge the urban/rural divide in practice and current capitalist urbanization patterns. Second, they call for conceptual diversity to question urban superiority and the prioritization of the urban over the rural. Third, they argue in favor of hybridizing both realms. Fourth, they suggest searching for performances that reconfigure the material, cultural, and power relations between the rural and the urban. In a similar spirit, Krahmer (2022), drawing on Massey's work on spatial relationality and theories of planetary urbanization, argues in favor of a "multiscalar degrowth" in "relational spaces". He attempts to pave the way towards studying degrowth urban/rural transformations under planetary urbanization by studying both conventional and alternative commodity geographies of fruit production in Chile and their commercial relations to the rest of the globe. In a different vein, Kallis et al. (2022) illustrate adeveloped Greek islands, examining how historical and contemporary human and cultural flows between islands and urban centres have shaped what they frame as "real-existing degrowth" geographies.

Our approach to degrowth spatial politics builds on these recent evolvements. It seeks to expand them by advancing a research agenda and methodology for conceptualizing and studying degrowth policies in diverse geographies and scales. Planetary urbanization allows for further conceptualizations, methods to explore it, and policies and politics to limit it. However, we don't see it as a "closure of the map", in line with some of its critics, in which cities have entirely absorbed the rural. We see it rather as an always incomplete tendency. The world, after all, is urbanizing; but many people still live in the countryside, along with their pluriversal ways of living and producing. The crucial task is to learn how to study these processes of uneven urbanization, to understand their conflicting directions, transformative dynamics, and weak points, and to suggest multiscalar strategies for undoing them with a view to a degrowth reorganization of space. This approach implies working at different levels. The first level is theoretical and conceptual; the only one with important advancements already. The second level involves empirical work on how material, economic, and cultural flows of planetary urbanization are altering concrete places globally, and how this affects the possibility of a pluriversal degrowth transition. The third and perhaps more demanding task is to explore the level of policies and politics of degrowth that can transform the negative impact of planetary urbanization while avoiding local or nationalistic parochialism and claustrophobic enclavism. In this sense, we argue that *de-urbanization for degrowth is not equal to relocalization* and doesn't necessarily point towards a spatial organization based on smallness and self-sufficient communities. Degrowth de-urbanization, on the contrary, should be considered as a multiscalar process that undoes the malaise of planetary urbanization to organize space in a multiplicity of forms and arrangements, many of which may not be radically different in shape from what they are today.

### Neglecting to appreciate the plural sources and futures of degrowth "nomadic utopianism"

A final remark on the sacrifice of the urban shift in the degrowth literature relates to the imaginary sources and the politics of degrowth. Degrowth has moved from a radical project aiming to destabilize post-political consensus and repoliticize discussions on sustainability and development, as previously explained, towards a credible alternative to the growth-based global development trajectory prominent in influential national and international venues. This evolution has shifted degrowth's research agenda towards more macroeconomic modeling and planning exercises, often articulated in universalistic ways. This is becoming an intensifying tension in the face of the climate emergency. The urban shift can be perfectly inscribed within this evolution that accepts urbanization as an ongoing and possibly irreversible trend (see remarks on depopulation policies in the previous section) while incorporating a more top-down planning as a credible strategy, among others.

We contend that this shift presents significant threats at the scientific, political, and imaginary levels for the future of degrowth despite apparent gains (i.e., the ability to enter mainstream discourses and to be taken "seriously" by policymakers, etc.). At the political level, the increased attention to the urban may affect the capacity of degrowth to remain pluriversal, anti-colonial, and grassroots-fueled. It has already been argued that a political economy of degrowth should avoid monolithic and orthodox realisms, because they could lead to technoscientific depoliticization and bypass—or even suppress—historical, spatial, and cultural differences (Chertkovskaya et al. 2019). If so, the revaluation of the rural in the degrowth scholarship is essential not only for the reasons presented in the previous subsections but also because it can decentralize and diversify the political project of a degrowth transition.

At the imaginary level, we think that degrowth has benefited and should continue to benefit from what has been framed as "nomadic utopianism," i.e., a political subjectivity built around the process of transition rather than on its described goal, which maximizes difference while creating new connections along the process (Chertkovskaya et al. 2019). Such an approach emphasizes the multiplicity and multiformity of degrowth, which is not only a moral imperative but also a vivid source of organizational and institutional innovation. Chertkovskaya et al. (2019) argue that the way for degrowth to become (counter)hegemonic does not pass from making degrowth a universalistic scientific paradigm but through the meaningful articulation of its multiple parts. We argue that the spatial expression of nomadic utopianism cannot be circumscribed within the ideal of the compact, green, smart, or 15-min city or any other spatial unit (be it a right-sized town, an ecovillage or else). Spatial justice presupposes cognitive justice (Paulson 2017), and a focus on the pluriverse of relational spaces can help us undo the rift between the urban and the rural and pursue a multiplicity of (inter)connected degrowth futures.

## Conclusion: towards a relational degrowth spatial politics

This paper explores the history of degrowth spatial politics from the early years of degrowth scholarship to today. We show how notions of space, place, planning, self-sufficiency, smallness, urbanization, and rural are used in different phases of this literature to invoke different meanings and to describe contrasting degrowth pathways. Furthermore, we examine the drivers of these conceptual and political shifts in the degrowth literature by assigning them to both external and internal causes. In the second section, we explore in detail how shifting all the focus to the urban realm may impact the radicality and relevance of the degrowth literature. In this concluding part, we attempt to systematize the direction for future studies.

The cornerstone of this effort is the thesis that degrowth spatial politics should embrace the rural in a multiscalar way that does not resort to simplistic representations of rural life and society or to calls for relocalisation to supposedly self-sufficient communities, villages, or small towns. We envisage degrowth spatial development as a process of undoing and restructuring the harmful spatial relations of planetary urbanization, depeasantization, and rural depopulation and abandonment to establish different, reciprocal, solidary, connections in a degrowth spirit between urban cores and rural peripheries, the Global North and the Global South. This is also a way in acknowledging the degree of difference and agency that places continue to demonstrate in opposition to planetary urbanization, as Katz (2021) suggests in her critique of planetary urbanization which she recognizes as important in overcoming cityism but blind to alternatives and oppositions in its totalizing approach. We argue that such a framework goes beyond calls for smart and green urban and rural spatial development, which we see as part of the problem and not as the solution. We argue that this theoretical framework can contribute to other theoretical accounts—of mainly Marxian origin—that call for countering uneven development but that fail to underpin the significance of economic growth as the main driver of these inequalities. Finally, we argue that such a framework can substantially boost the already rich discussions recently developed in the field of degrowth spatial politics.

A research agenda on the undoing of planetary urbanization as a contribution to this degrowth spatial politics, needs to be a both theoretical and empirical project. It should include both a re-interpretation of processes of planetary urbanization through a degrowth lens and re-interpret those alternatives of real-existing degrowth not anymore in a logic of essentialized spatial forms but rather in a perspective of processes, tendencies and relationalities. In addition, it will be necessary to enlarge the number of practices studied in this perspective, widening the empirical agenda. This, finally, needs to include a methodological discussion, too, about how to study such complex, often contradictory and intertwined processes in which it is not always so easy to separate what is the problem from what is the alternative, precisely because we no longer draw rigid boundaries around our objects of research.

## Footnotes

1 i.e. A type of utopia, usually an enclave, in which spatial form controls temporality, social change, and history to ensure internal harmony and happiness.

2 REAL, Decycle, Prospera.

3 XXX.

4 Referred also to in Arboleda (2020)

5 In Arboleda (2020)

6 https://www.oecd.org/content/dam/oecd/en/topics/policy-sub-issues/demographic-change-in-regions/Flyer%20-%20OECD%20Shrinking%20Smartly%20and%20Sustainably.pdf
